# Prognostic factors in infective endocarditis in general hospitals in the Netherlands

**DOI:** 10.1007/s12471-016-0846-2

**Published:** 2016-05-17

**Authors:** F. van den Brink, J. Hasenaar, V. Winia, M. Klomp, B. Van Vlies, D. Nicastia, B. Groenmeijer, R. Braam, W. Jaarsma, A. J. Funke Kupper

**Affiliations:** 1 Department of Cardiology, St Antonius Hospital, Koekoekslaan 1, 3435 CM Nieuwegein, The Netherlands; 2 Department of Anesthesiology, Leiden University Medical Center, Leiden, The Netherlands; 3 Department of Cardiology, Leiden University Medical Center, Leiden, The Netherlands; 4 Department of Cardiology, Kennemer Gasthuis, Haarlem, The Netherlands; 5 Department of Cardiology, Gelre Hospital, Apeldoorn, The Netherlands

**Keywords:** Endocarditis, Survival analysis, Hospitals, general, Mortality, Morbidity, Staphylococcus aureus

## Abstract

**Introduction:**

Despite advances in treatment, infective endocarditis (IE) still ranks amongst the most lethal infectious diseases. We sought to determine prognostic factors in general hospitals in the Netherlands as research in this setting is scarce.

**Results:**

Between 2004 and 2011, we identified 216 cases of IE, 30.1 % of which were prosthetic valve IE. This leads to an annual incidence of IE of 5.7 new cases per 100,000 persons per year. Women were less likely to undergo surgical intervention (OR = 1.96, 95 % CI 1.06–3.61, *p* = 0.031). Also, ageing was an independent prognostic factor for not receiving surgery in a multivariate analysis (annual OR = 1.04, 95 % CI 1.02–1.06, *p* < 0.001). Female sex was a prognostic factor for mortality (OR = 2.35, 95 % CI 1.29–4.28, *p* = 0.005). Age was also an independent prognostic factor for mortality (OR = 1.05, 95% CI 1.03–1.08, *p* < 0.001). Conservative treatment was a prognostic factor for mortality (OR = 3.39, 95 % CI 1.80–6.38, *p* < 0.001) whereas surgical intervention was an independent prognostic factor for adverse events (OR = 3.03, 95% CI 1.64–5.55, *p* < 0.001). *Staphylococcus aureus* was an independent prognostic factor for adverse events (OR = 2.05, 95 % CI 1.10–3.84, *p* = 0.024) but not for mortality.

**Conclusion:**

This study shows that endocarditis in general hospitals has a high rate of morbidity and mortality. Even when treated, it ranks as one of the most lethal infectious diseases in the Netherlands, especially in women and the elderly.

## Introduction

Since William Osler first described ‘malignant endocarditis’ in 1885, there has been an evolution in the pathophysiology and treatment of this rare but often lethal disease [1–4].

A decreased incidence of rheumatic heart valve disease but increased incidence of degenerative heart valve disease has changed the demographics of patients affected with infective endocarditis (IE) in the Western world [5–8]. Interestingly, in the Netherlands, due to lower numbers of intravenous drug users, the frequency of right-sided endocarditis has notably dropped [9]. At the same time, the worldwide rise in methicillin-resistant *Staphylococcus aureus* (MRSA) has augmented MRSA-related IE [10–14]. Furthermore, there has been a substantial increase in the use of prosthetic heart valves, both mechanical and biological [15]. Also, the increased use of devices such as pacemakers and implantable cardiac defibrillators is posing new challenges [10].

Previous studies in the Netherlands that assess the treatment of IE enrolled patients who were treated in a tertiary hospital [16–19]. One can hypothesise that patient demographics and outcome between a general and a tertiary hospital differ due to patient selection and available treatment modalities. Our experience is that an IE population in a tertiary centre will largely consist of patients eligible for surgical intervention or those who have undergone an operation. There will be very few patients who receive conservative treatment. A general hospital’s population, on the other hand, will mainly consist of patients who receive conservative treatment and a few patients recovering from an earlier operation. However, studies focussing on IE in general hospitals are scarce [20]. The primary aim of our study is to describe the demographics and identify the prognostic factors of IE in general hospitals in the Netherlands.

Furthermore, a changing insight into the demography and prophylaxis of IE [16, 17] has led to the development of the 2009 European Society of Cardiology (ESC) guideline on prevention, diagnosis and treatment of IE [21]. The result of this guideline has been a less strict use of prophylaxis. The secondary aim of this study is to evaluate implementation of the revised guideline on the prevention of IE in our patient population [21].

## Material and Methods

A multicentre retrospective cohort study was performed in three general hospitals in the Netherlands (Spaarne [former Kennemer] Gasthuis Haarlem, Gelre Hospital Apeldoorn, Gelre Hospital Zutphen). Data collection was performed between 2012 and 2013. Patients treated for IE were identified using the Dutch national in-hospital insurance registry (Diagnose Behandel Combinatie). All patients treated for definite or possible IE between 2004 and 2011 were included. All patient charts, echocardiogram reports and microbiology reports were reviewed. Median follow-up was 4.2 years (0.3–8.0). The modified Duke criteria were applied to identify patients with definite IE [21]. Patients with possible IE, according to the modified Duke criteria, but treated as definite IE were also included. Patients with native and/or prosthetic valve IE were enrolled as well as pacemaker device and/or lead infection. The treating cardiologist evaluated the transthoracic and transoesophageal echocardiograms and identified the valve or lead vegetation. MRSA was defined as such after consultation with a microbiologist or when another antibiotic was used than the first-line therapy according to the local guidelines.

Mortality was defined as all-cause mortality within the follow-up period. Adverse events were defined as IE-related adverse events during hospitalisation requiring medical intervention or prolonging hospital stay. The adverse events included were infarction (including stroke and peripheral septic emboli), bleeding, recurrent endocarditis, tachyarrhythmias and abscess formation at any location.

Statistical analysis was performed using SPSS (I.B.M. Armonk, NY, USA) and R (www.r-project.org). Student’s t test was used for continuous variables and Fisher’s exact test/Chi-square test for categorical variables. Multivariate logistic regression analyses were performed to determine independent risk factors for mortality and morbidity. A two-tailed *p* value of *p* < 0.05 or less was considered statistically significant.

As this is a retrospective study, approval of the local ethics committee was not needed.

## Results

### Patient characteristics

**Fig. 1:**
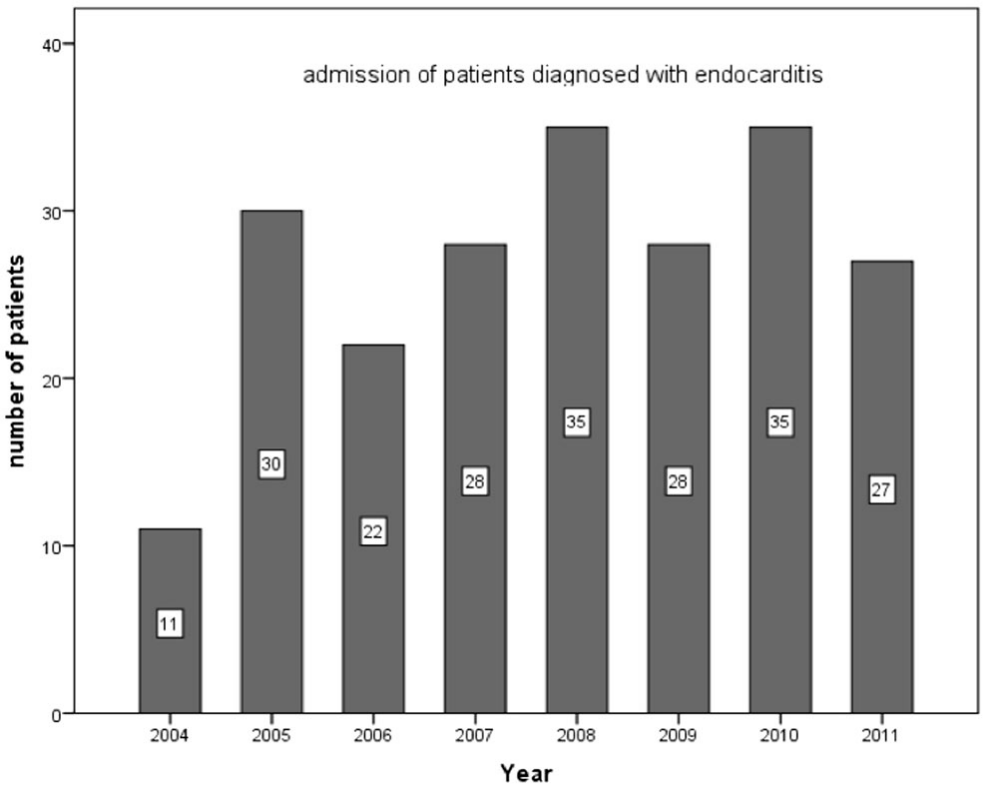
Annual distribution of new IE cases per year

Between 2004 and 2011, we identified 216 cases of IE (Fig. 1). Based on the three general hospitals and the per-hospital catchment area, this would amount to an annual incidence of IE of 5.7 new cases per 100,000 persons per year [22, 23]. The mean age at the time of the diagnosis was 67.5 years (22–97). Men were more affected than women: 62.5 % versus 37.5 %. Definite IE was diagnosed in 82.8 % of the patients. Transthoracic echocardiogram confirmed the diagnosis in 19.4 % of the cases and transoesophageal echocardiogram in 74.1 %. In 6.5 % no vegetation was visible. A total of 44 % of the population did not have a predisposing risk factor for IE.

### Affected valves

One-third of the patients had prosthetic valve IE (30.1 %). A pacemaker device lead located at the right side of the heart was affected in 17 cases. In these 17 cases a left-sided heart valve was also involved in 2 patients. Only 10 patients had tricuspid or pulmonary valve IE only, none of which were related to a pacemaker lead infection.

### Microbiology

Positive blood cultures were found in 90.7 % of the patients. A total of 30.1 % of all patients had *Staphylococcus aureus* in 1 or more blood cultures, making it the most prevalent microorganism. There was only 1 patient with a MRSA positive culture. A bacterial access location was found in 115/216 (53.2 %) of the patients. The most prevalent access locations were oropharyngeal (30/115, 26.0 %) cutaneous (23/115 20.0 %), urinary tract (12/115 10.4 %) and the colon (10/115, 8.7 %). There were only 2 patients with intravenous drug use as a cause of their IE.

### Surgical versus conservative treatment

A total of 84 (38.9 %) patients were accepted for surgical intervention. Eight underwent only pacemaker lead extraction. *S. aureus* endocarditis or prosthetic valve IE were not independent prognostic factors for receiving surgical intervention. Women were less likely to undergo a surgical intervention (OR = 1.96, 95 % CI 1.06–3.61, *p* = 0.031). Also, ageing was an independent prognostic factor for not receiving surgery in a multivariate analysis (annual OR = 1.04, 95 % CI 1.02–1.06, *p* < 0.001) (Tab. 1).

**Tab. 1:** Prognostic factors for conservative treatment, multivariate analysis

Female sex	OR = 1.96, 95 % CI 1.06–3.61, *p* = 0.031
Age (per life year)	OR = 1.04, 95 % CI 1.02–1.06, *p* < 0.001

### Outcomes

All-cause mortality was 36.1 %. Mortality in women was significantly higher than in men (49.3 % versus 28.2 %, *p* = 0.002). Female sex was an independent prognostic factor for mortality (OR = 2.35, 95 % CI 1.29–4.28, *p* = 0.005). The mean age at time of death was 76 years while the mean age for the surviving patients was 65 (*p* < 0.001, 95 % CI 4.66–12.15). Age was an independent prognostic factor for mortality as well (OR = 1.05, 95% CI 1.03–1.08, *p* < 0.001) (Tab. 2). Age and gender were not independent prognostic factors for adverse events.

**Tab. 2:** Prognostic factors for mortality, multivariate analysis

Age (per life year)	OR = 1.05, 95% CI 1.03–1.08, *p* < 0.001
Female sex	OR = 2.35, 95 % CI 1.29–4.28, *p* = 0.005
Conservative treatment	OR = 3.39, 95 % CI 1.80–6.38, *p* < 0.001

*S. aureus* was an independent prognostic factor for adverse events (OR = 2.05, 95 % CI 1.10–3.84, *p* = 0.024) but not for mortality (OR = 1.90, 95 % CI 0.99–3.66, *p* = 0.054) (Tab. 3).

**Tab. 3:** Prognostic factors for adverse events, multivariate analysis

*Staphylococcus aureus* infection	OR = 2.05, 95 % CI 1.10–3.84, *p* = 0.024
Surgical treatment	OR = 3.03, 95% CI 1.64–5.55, *p* < 0.001

Mortality was higher in prosthetic valve IE 42/65 (66.2 %) when compared with native valve IE 56/151 (37.0 %) (*p* < 0.001). In a multivariate analysis between prosthetic valve IE versus native valve IE there were no significant differences in mortality and adverse events. Mortality was higher in the conservative treatment group 62/132 (46.9 %) when compared with the surgical group; 16/84 (19.0 %) (*p* = <0.001). Conservative treatment was an independent prognostic factor for mortality (OR = 3.39, 95 % CI 1.80–6.38, *p* < 0.001) (Tab. 2). The incidence of adverse events was higher in the surgical group (62/84, 73.8 %) than in the conservative treatment group (64/132, 48.4 %). Surgical intervention was an independent prognostic factor for adverse events (OR = 3.03, 95% CI 1.64–5.55, *p* < 0.001) (Tab. 3).

### Introduction of new ESC guideline

Our study includes 126 patients before and 90 patients after the introduction of the new ESC guideline on the prevention, diagnosis and treatment of IE. Amongst these patients, we did not find an evident increase in incidence of IE before and after the introduction of the new ESC guideline. Comparing the population before and after introduction of the new guideline there was no difference in mortality (OR = 1.82, 95 % CI 0.97–3.50, *p* = 0.0665) or the number of adverse events (OR = 0.60, 95 % CI 0.34–1.04, *p* = 0.0698). There was no significant difference in the chance of receiving a surgical intervention before and after the introduction of the ESC guideline. Of the whole cohort only 1/216 (0.46 %) patient had received prophylaxis.

## Discussion

To our knowledge this is the first multicentre retrospective cohort study on IE performed only in general hospitals in the Netherlands. This research shows that overall mortality associated with IE is higher than in other recent studies [16–19]. As there is no selection bias due to referral for possible surgical intervention we believe that, despite improvements in treatment, mortality still remains high and makes IE one of the most lethal infectious diseases in the Western world.

Although this study focuses on all-cause mortality it does show there might be an underestimation of IE-related mortality based on the rates from previous studies [16–19]. As stated in our introduction, patient demographics and outcomes between general hospitals and tertiary hospitals differ: more patients who are not eligible for operation will remain in a general hospital and will receive conservative treatment. Our study shows that conservative treatment is related to a higher mortality, hence a possible explanation for the high overall mortality rates. Female sex is a prognostic factor for mortality but not for adverse events. Our study shows that women are less likely to receive surgical intervention. As this study also shows that surgical intervention has a better prognosis, this may be an explanation why mortality in women is higher than in men. Earlier surgical intervention may be the key to improving survival in women with IE. Previous studies endorse our results, stating that surgical intervention improves survival and adverse event-free interval [2, 24].

Prosthetic valve IE has a higher mortality when compared with native valve IE, but our study does not show a difference in adverse events between prosthetic and native valves. This might be explained by the population in general hospitals in which patients will not be eligible for surgical intervention due to existing comorbidities and therefore will not receive surgery. As surgery is no longer an option, they may have entered a palliative setting in which there was no further recording of adverse events and as such a possible loss to follow-up. A similar mechanism may explain the difference in mortality and adverse events between surgical treatment and conservative treatment. Patients who received surgery possibly had a better preoperative physical condition and therefore had higher survival rates. As the monitoring postoperatively might be better when compared with conservative treatment, there could have been an earlier detection of adverse events. Patients who received conservative treatment may have entered a palliative setting in which there was no further recording of adverse events.

*S. aureus* IE increases the risk of disease-related morbidity. As *S. aureus* spread is increasing in the population, this is a growing concern and may lead to a higher disease burden in the future. Fortunately, MRSA does not yet pose a significant problem in IE. The fact that MRSA has hardly penetrated the endocarditis population does not mean that it will not do so in the future. In a number of cases in our cohort no organism was cultured. This might be due to antibiotics having been started by a general practitioner prior to presentation in a hospital.

Although we do not see a difference in the number of new cases, mortality, adverse events and number of surgical interventions between the era before and after the introduction of the new ESC guidelines, this may be due to the small sample size. Other recent studies have shown an increase in incidence in IE related to the introduction of the National Institute of Clinical Excellence 2008 guideline and the America College of Cardiologist/American Heart Association 2007 guidelines [25, 26].We expect the Dutch population to be similar to the ones described by Pant et al. and Dayer et al. and think a similar change in the prevalence of endocarditis might have taken place in the Netherlands since the introduction of the guidelines.

The study design, a multicentre retrospective cohort study, is a limitation to this study. The difference in time of follow-up of the patients included varies widely and as such might have influenced the numbers for mortality and adverse effects.

## Conclusion

This research shows that the endocarditis in general hospitals has a high rate of morbidity and mortality. Even when treated it ranks as one of the most lethal infectious diseases in the Netherlands, especially in women and the elderly. Further investigation is needed to determine optimal treatment and the effectiveness of the new ESC Guideline on the prevention, diagnosis and treatment of infective endocarditis.
